# Pet Ownership and Its Influence on Animal Welfare Attitudes and Consumption Intentions Among Chinese University Students

**DOI:** 10.3390/ani14223242

**Published:** 2024-11-12

**Authors:** Yaoming Liang, Chengmin Meng, Ruiqi Chen, Yongkun Yang, Yonghui Zeng

**Affiliations:** 1 College of Economics & Management, South China Agricultural University, Guangzhou 510642, China; evanliang@scau.edu.cn (Y.L.); ricky_ccc@163.com (R.C.); 2 College of Electronic Engineering & College of Artificial Intelligence, South China Agricultural University, Guangzhou 510642, China; 3 Party and Government Office (Research Office), South China Agricultural University, Guangzhou 510642, China; mengcm@scau.edu.cn; 4 Institute of Bast Fiber Crops, Chinese Academy of Agricultural Sciences, Changsha 410006, China; 5 College of Public Management, South China Agricultural University, Guangzhou 510642, China

**Keywords:** pet ownership, animal welfare attitudes, consumption intentions, propensity score matching (PSM), Chinese university students

## Abstract

As global awareness of animal welfare continues to rise, it has become essential to understand the factors that shape individual attitudes and consumption behaviors related to animal welfare. This study empirically investigates how pet ownership influences attitudes towards animal welfare and related consumption intentions among Chinese university students. Findings demonstrate that students from pet-owning households exhibit significantly more favorable attitudes and behaviors concerning animal empathy, awareness of animal welfare, willingness to purchase animal welfare certified products, and the willingness to pay a premium for animal welfare labels. Pet ownership fosters emotional resonance, enhancing empathy and perceptions regarding animal welfare, and influences preferences for animal welfare certified products through simulated consumer choices. By applying propensity score matching (PSM) to mitigate endogeneity concerns, this research advances theoretical discussions surrounding animal welfare attitudes and consumption behaviors. Looking ahead, the promotion of animal welfare should involve coordinated efforts across educational institutions, policy frameworks, and market mechanisms to cultivate a broader understanding and adoption of animal welfare principles.

## Simple Summary

This study explores how having pets influences Chinese university students’ attitudes and willingness to buy animal welfare certified products. It finds that students from households with pets are more empathetic toward animals and show a greater willingness to support animal welfare certified products, even if it means paying a premium. The research highlights the important role of pet ownership in shaping ethical attitudes toward animals and suggests that this background could be valuable for promoting animal welfare more broadly across society. Additionally, the study emphasizes that policies, businesses, and education should work together to raise awareness about animal welfare and encourage ethical consumption.

## 1. Introduction

In recent years, animal welfare has increasingly emerged as a critical indicator of social progress and sustainable development [1,2]. In 2022, the United Nations Environment Assembly adopted a landmark resolution recognizing the relationship between animal welfare and environmental sustainability, marking the first time that animal welfare was formally integrated into the global environmental agenda. This development underscores the growing international consensus on the strategic importance of animal welfare, positioning it as a vital measure of national modernization [2]. Beyond its direct benefits in production systems, animal welfare also delivers broader societal advantages, including improved workforce well-being, competitive business gains, and reduced health risks for both humans and animals [3,4,5]. Furthermore, it plays a pivotal role in mitigating the impacts of climate change [6]. Within this global context, animal welfare has ignited extensive ethical and social discourse, particularly in relation to consumer behavior. As markets expand, consumer attitudes and purchasing behaviors have become significant drivers in the promotion of animal welfare certified products [7,8]. Understanding the factors that shape consumer attitudes and behaviors towards these products is crucial for advancing animal welfare policies and promoting ethical consumption practices.

Despite the increasing global emphasis on animal welfare certified products, much of the existing research has predominantly focused on developed Western countries, particularly within their established market environments. International studies consistently highlight the crucial role that moral awareness and societal values play in shaping consumer attitudes and behaviors related to animal welfare [9,10,11,12,13]. In these developed nations, markets for animal welfare certified products are relatively mature, and consumer awareness is well-formed, with many individuals aligning their ethical beliefs with purchasing behaviors. However, in China—where the market for animal welfare certified products is still in its infancy—consumer awareness of and attitudes toward animal welfare are less developed, and the concept itself remains relatively novel [1]. While a substantial body of literature has examined the correlation between animal welfare attitudes and consumption behaviors, there remains a gap in understanding the specific factors influencing these attitudes and behaviors within China’s unique cultural context. Investigating the external influences on consumer attitudes toward animal welfare in China, particularly among younger consumers, presents an important research avenue.

University students, as a key demographic in the future consumer market, hold a pivotal role in shaping emerging trends, particularly in sectors such as animal welfare certified products [14,15,16]. Their consumption patterns not only mirror evolving societal values but also have the potential to guide broader market directions. With their higher education levels and better access to information, university students are often at the forefront of adopting new ideas, including ethical consumption. Understanding their attitudes and intentions toward animal welfare offers critical insights for predicting shifts in consumer behavior, making them a valuable group for studying the future trajectory of animal welfare in China.

Pet ownership has emerged as one such influencing factor, which has garnered increased attention in recent years. Interactions with pets can strengthen an individual’s emotional connection with animals and enhance their awareness of animal welfare [17,18,19,20,21]. This combined effect on both emotional and perceptual dimensions not only manifests in the care of pets but may also extend to attitudes toward other animals, influencing moral inclinations and consumer behavior [22,23]. Research indicates that pet owners are generally more attuned to animal welfare concerns [21,24], exhibit a greater sensitivity to ethical considerations and social responsibilities, and show an increased willingness to purchase products that support animal welfare [25,26]. While previous research has explored the correlation between pet ownership and animal welfare attitudes, causal relationships remain underexplored, especially in China. This study aims to fill this gap by employing propensity score matching (PSM) to examine the impact of pet ownership on attitudes toward animal welfare and consumption intentions within the Chinese cultural context.

Following this context, the central objective of this study is to examine the extent to which pet ownership influences individual attitudes toward animal welfare and consumption intentions. In particular, this research focuses on university students’ attitudes, exploring the dual dimensions of empathy (emotional) and perceptions of animal welfare (perceptual). With the rise of animal welfare awareness in China, understanding the factors influencing the consumption of animal welfare certified products is essential. As university students are poised to become the future drivers of the animal welfare market, their perspectives provide unique insights into future trends [14,15,16]. By utilizing survey data from university students and applying PSM to reduce selection bias, this study provides a more robust analysis of how pet ownership influences attitudes and consumption intentions, offering empirical insights into the relationship between these variables within China’s unique social and economic environment.

The contributions of this paper are threefold: First, it systematically investigates the influence of pet ownership on attitudes toward animal welfare and consumption intentions within the Chinese context, thereby expanding the geographical and cultural scope of existing research. Second, by utilizing PSM, the study addresses the issue of self-selection bias, improving the causal interpretability of the findings. Finally, the research explores not only the effect of pet ownership on attitudes but also its influence on actual consumer behaviors, such as purchase intentions and willingness to pay a premium, thereby offering a more holistic perspective on the topic.

The structure of this paper is as follows: Section 2 reviews the literature on animal welfare and presents the research hypotheses; Section 3 describes the data sources, variable definitions, and research methodology; Section 4 presents the empirical analysis results; Section 5 discusses the findings and provides recommendations; and Section 6 concludes with the study’s key findings.

## 2. Literature Review and Research Hypotheses

### 2.1. Pet Ownership and Attitudes Toward Animal Welfare

Attitudes toward animal welfare are shaped by a range of factors, including cultural values, education, and personal experiences with animals, such as pet ownership. Among these factors, pet ownership has been found to significantly influence both the emotional and perceptual dimensions of individuals’ attitudes toward animal welfare [27,28]. While this study focuses on pet ownership, it is important to recognize that other factors also contribute to shaping these attitudes [29,30,31]. The goal here is to examine pet ownership as one potential influencing factor within the broader context of animal welfare attitudes.

The “pets as ambassadors hypothesis” suggests that daily interactions with pets not only deepen emotional attachments but also foster moral concern for other animal species, enhancing awareness of animal welfare more broadly [22,23]. These interactions cultivate empathy, defined as the ability to perceive and respond to the emotional states of others. Research shows that pet owners are more likely to demonstrate empathy toward animals, as pet ownership fosters sensitivity to animal suffering and well-being [17,23,32]. This increased empathy often leads to greater awareness of animal welfare issues, motivating consumers to make ethically conscious decisions regarding their product choices, particularly those related to animal welfare [33].

From a perceptual perspective, pet ownership influences individuals’ attitudes by shaping their interpretations and views of animal welfare issues. Specifically, perceptions of animal welfare encompass individuals’ subjective views on the ethical and practical considerations surrounding the treatment and welfare of animals. Pet owners may be more likely to perceive animal welfare as an important societal concern [34,35], which informs their broader attitudes toward ethical consumption and animal welfare practices. These perceptions, shaped by personal experiences and societal influences, may affect how individuals assess the importance of supporting animal welfare through their everyday decisions [36].

In addition to emotional and perceptual influences, socioeconomic characteristics also play a role in shaping the relationship between pet ownership and attitudes toward animal welfare. Research suggests that younger, urban, and better-educated individuals are more likely to own pets and be attuned to animal welfare concerns [28,37]. As such, while pet ownership may enhance both emotional and perceptual pathways, the strength of this influence may vary across different demographic groups.

Based on this review, the following hypotheses are proposed. H1a and H1b represent two key dimensions of attitudes toward animal welfare. While H1a focuses on the emotional response (empathy) to animal suffering, H1b addresses the perceptual understanding of animal welfare issues. Together, these two dimensions provide a comprehensive view of how pet ownership influences both the emotional and perceptual aspects of attitudes toward animal welfare:

**H1.**  *Pet ownership has a significant positive impact on attitudes toward animal welfare.*

**H1a.**  *Pet ownership positively influences animal empathy.*

**H1b.**  *Pet ownership positively influences perceptions of animal welfare.*

### 2.2. Pet Ownership and Animal Welfare Consumption Intentions

Consumer behavior regarding animal welfare certified products has garnered significant attention in recent years [13], particularly as younger consumers increasingly seek products aligned with ethical practices and animal welfare standards [16]. Research suggests that pet ownership strengthens emotional connections with animals [23,24], and that these bonds may directly shape consumer preferences and behaviors toward animal welfare certified products [16,38]. Through their experience with pets, individuals may develop a heightened awareness of animal welfare, which could influence their purchasing decisions, particularly when evaluating animal welfare certified products [1].

Purchase intention refers to a consumer’s likelihood of choosing to buy products that align with animal welfare standards [1]. Studies have consistently shown that pet owners are more inclined to purchase products with animal welfare certifications due to the stronger emotional bonds they develop with animals [25,26,35]. This suggests that pet ownership fosters an increased likelihood of supporting animal welfare products, indicating that pet owners are more likely to integrate animal welfare considerations into their purchasing decisions [39]. While purchase intention reflects a positive behavioral inclination, it also indicates how closely animal welfare values are integrated into the consumer’s broader value system.

Similarly, willingness to pay a premium reflects consumers’ recognition of the ethical value embedded in animal welfare certified products and their readiness to pay more for such goods [13,16]. Pet ownership has been shown to significantly enhance this willingness, as pet owners are generally more attuned to the ethical dimensions of product choices. Although practical factors such as financial capacity and product availability may influence actual purchasing behavior, willingness to pay a premium reflects a deeper ethical commitment to animal welfare values, which often results in a higher likelihood of paying more for ethically certified products [13,40].

Additionally, socioeconomic factors such as education, age, and urban living further moderate the relationship between pet ownership and consumer behavior. Younger, better-educated, and urban-dwelling individuals not only tend to be more attuned to animal welfare issues but also often have greater access to welfare-certified products and the resources to purchase them, which may further support their willingness to pay a premium for these products [16,39,41,42]. These factors suggest that pet ownership enhances both purchase intention and willingness to pay a premium across different consumer demographics, albeit with varying levels of influence depending on socioeconomic characteristics.

Based on the literature and the factors discussed, we propose the following hypotheses to explore the relationship between pet ownership and animal welfare consumption intentions. Purchase intention and willingness to pay a premium reflect complementary aspects of consumer behavior. Purchase intention refers to a general willingness to buy animal welfare products, whereas willingness to pay a premium indicates a deeper commitment, where consumers are ready to invest financially in products certified for animal welfare. By considering both dimensions, we aim to capture the full range of consumer intentions, from general ethical support to specific financial commitments:

**H2.**  *Pet ownership has a significant positive impact on animal welfare consumption intentions.*

**H2a.**  *Pet ownership positively influences the willingness to purchase animal welfare certified products.*

**H2b.**  *Pet ownership positively influences the willingness to pay a premium for animal welfare certified products.*

## 3. Data, Variables, and Methods

### 3.1. Data

Data were collected using an online questionnaire, distributed via the “Wenjuanxing” platform, a leading survey platform in China. The questionnaire captured key variables such as demographic characteristics, family background, pet ownership, and attitudes toward animal welfare and consumption intentions. It underwent several rounds of expert review and revisions to ensure scientific validity and clarity. A pilot test with 30 university students in March 2023 helped refine the questionnaire’s wording, structure, and logic.

The formal survey was conducted between July and August 2023, targeting university students in Guangdong Province, a region known for its economic development and openness to global ideas. These students represent a forward-looking demographic, particularly in emerging sectors like animal welfare products. A total of 1409 responses were collected, incentivized by a random red packet reward ranging from CNY 1 to 3. After data cleaning, which involved excluding incomplete or inconsistent responses, 1140 valid questionnaires were retained, yielding an effective response rate of 80.91%.

### 3.2. Variable Description

#### 3.2.1. Dependent Variables

This study employs four specific indicators to measure university students’ attitudes toward animal welfare and their behavioral intentions. Attitudes are assessed through two dimensions: animal empathy and perceptions of animal welfare; behavioral intentions are captured using two indicators: willingness to purchase animal welfare certified products and willingness to pay a premium for animal welfare labels.

(1) Animal Empathy

Animal empathy reflects the respondent’s emotional reaction to animal suffering. The questionnaire included the following statement: “Please evaluate the following statement: ‘I feel upset every time I see animals being abused or in pain’”. Responses were measured on a 5-point Likert scale, ranging from “1 = Strongly Disagree” to “5 = Strongly Agree”. Based on the survey results, the average score for animal empathy was 4.061 (with a standard deviation of 0.952).

Given the distribution of responses, where the majority of students scored either 4 or 5, we classified animal empathy into three distinct categories to better capture the variability in empathy levels. Respondents scoring 1 to 3 were classified as having low empathy, those scoring 4 as having moderate empathy, and those scoring 5 as having high empathy. This three-category classification allows for a more nuanced analysis of empathy levels, ensuring that subtle differences between moderate and high empathy are preserved. The following analysis uses these three categories to examine the relationship between pet ownership and animal empathy.

(2) Perceptions of Animal Welfare

Perceptions of animal welfare reflect respondents’ subjective views and interpretations regarding key animal welfare issues. Four statements were used to assess these perceptions, each capturing distinct dimensions of ongoing debates about animal welfare in Chinese society: “Farm animal welfare does not need to be considered since they are eventually slaughtered for food”, “Human welfare has not yet been fully met, so it’s not time to consider animal welfare”, “The cost of welfare farming is too high and unsuitable for our country’s reality”, and “Some welfare farming measures are merely commercial gimmicks or a result of curiosity from farmers”.

These statements were selected based on prevailing discussions and misconceptions about animal welfare that we have frequently observed in our broader research on the topic. In their study, Liang et al. [16] identified similar themes when investigating perceptions of animal welfare among Chinese university students. These statements were designed to capture key areas of debate, including the ethical trade-offs between human and animal welfare, economic considerations, and skepticism toward commercialized welfare practices. This reflects a range of attitudes commonly encountered in Chinese society, where animal welfare is still an emerging concept.

Each statement was evaluated on a 5-point Likert scale, ranging from “1 = Strongly Disagree” to “5 = Strongly Agree”. The Cronbach’s α coefficient for each statement exceeded 0.75, with an overall reliability of 0.848, indicating strong internal consistency for the scale. The average total score across these statements was 2.680 (with a standard deviation of 0.842). For analytical purposes, we classified respondents’ perceptions of animal welfare into two levels: high and low. A ”high” perception reflects a more favorable and supportive attitude toward animal welfare, indicating a belief that animals merit ethical consideration and humane treatment, despite potential practical challenges. Conversely, a “low” perception suggests a more utilitarian or pragmatic outlook, with respondents potentially prioritizing human needs over animal welfare concerns. Respondents scoring below the mean were classified as having high perceptions of animal welfare (assigned a value of 1), while those scoring above the mean were classified as having low perceptions (assigned a value of 0). This classification enables an examination of how variations in perceptions align with differing ethical perspectives on animal welfare within the study’s framework.

(3) Willingness to Purchase Animal Welfare Certified Products

Willingness to purchase animal welfare certified products measures respondents’ intentions toward consuming specific animal welfare certified products. To ensure representativeness and prompt respondents to make concrete consumption decisions, this study selected “milk” as the target product, given its status as a common, affordable item frequently consumed by university students. The survey posed the question: “If animal welfare-certified milk were available on the market, would you be willing to buy it?” Responses were measured on a 5-point Likert scale, ranging from “1 = Definitely Not” to “5 = Definitely Yes”. The statistical results indicated an average score of 3.250 (with a standard deviation of 0.650). For analytical purposes, respondents with scores above the mean were classified as having high willingness to purchase and assigned a value of 1, while those scoring below the mean were classified as having low willingness to purchase and assigned a value of 0.

(4) Willingness to Pay a Premium for Animal Welfare Labels

To measure respondents’ willingness to pay a premium for animal welfare certified products, this study employed a discrete choice experiment (DCE) focused on milk consumption. Several attribute variables were included, such as brand, animal welfare label, protein content, shelf life, and price. The experiment presented different product combinations, prompting respondents to select their preferred option, allowing for the derivation of their willingness to pay a premium for animal welfare certified products. A mixed logit model was used to analyze the data and estimate the willingness to pay (WTP) for animal welfare labels among the university students surveyed. Following the approach by Liu and Wang [43], individual-level coefficients for the animal welfare label premium were extracted. The statistical results showed that the average willingness to pay a premium for animal welfare labels among university students was CNY 3.617 (with a standard deviation of 0.960). Further details on the experimental design and statistical methods are elaborated in a related study [16].

#### 3.2.2. Core Independent Variable

The core independent variable in this study is “pet ownership”, which broadly refers to the presence of pets in the household. It was measured through a specific survey question, “Does your family keep pets?”, with response options of “1 = Yes” and “0 = No”. While this question does not capture detailed aspects of pet ownership, such as the respondent’s caretaking responsibilities, the type of pet, or the duration of pet ownership, it serves as a proxy for pet-related experiences that may influence respondents’ attitudes toward animal welfare and their consumption intentions. Previous research indicates that simply growing up in a household with pets can positively affect individuals’ empathy towards animals and enhance their understanding of animal welfare issues [1,19,44]. Given the scope of our study, this measure provides a useful indicator for exploring the potential relationship between pet ownership and ethical attitudes and behaviors related to animal welfare.

#### 3.2.3. Control Variables

To minimize the influence of extraneous factors on the research outcomes, a series of individual- and family-level control variables were selected. These control variables include gender, age, BMI (body mass index), academic major, monthly living expenses, and the number of permanent family members. The selection of these variables is grounded in theoretical considerations, as previous studies suggest they may affect respondents’ attitudes and consumption behaviors related to animal welfare [1,4,16,45,46]. The specific definitions and operationalization of each variable are detailed in Table 1.

### 3.3. Analytical Methods and Steps

The core objective of this study is to determine whether “pet ownership” significantly influences Chinese university students’ attitudes toward animal welfare and their consumption intentions. Since the distribution of “pet ownership” is not random and may be influenced by factors such as gender, economic conditions, and academic background, there is potential for selection bias within the sample. This selection bias could lead to systematic differences between the treatment group (students from households with pets) and the control group (students from households without pets), thus compromising the accuracy of causal inferences.

To address this issue, the study employs the PSM method. PSM, grounded in the counterfactual framework, aims to construct a treatment group and a control group that are comparable in terms of covariates, thereby mitigating systematic differences between samples and enabling a more accurate estimation of the causal effects of “pet ownership” on attitudes toward animal welfare and consumption intentions. Specifically, this study matches students from households with pets to those from households without pets based on individual characteristics such as gender, age, BMI, academic major, monthly living expenses, and the number of permanent family members.

The matching process involves the following steps:

(1) Estimating Propensity Scores: First, a logistic regression model is used to estimate the probability of each sample individual coming from a household with pets, which generates the propensity score P(x) = Pr(T = 1∣X), where T indicates whether the individual comes from a household with pets and X represents the matrix of covariates. Assuming no unobserved confounding and that the common support condition holds, PSM can achieve effects similar to covariate matching.

(2) Selecting Matching Methods: To ensure the robustness of the results, this study employs three mainstream matching methods: ① Kernel Matching: This method utilizes all control group samples for weighted matching, with weights assigned based on the differences in propensity scores between treatment and control samples. The default kernel function and bandwidth parameters are used in this study. ② Radius Matching: Also known as caliper matching, this method restricts the absolute distance between propensity scores to control the precision of sample matching. The caliper range is set to 0.01. ③ Nearest Neighbor Matching: For each treatment group sample, the control group sample with the closest propensity score is matched. A caliper range of 0.01 is adopted and k = 4 is set, meaning each treatment group sample is matched with four control group samples to minimize the mean squared error.

(3) Estimating Causal Effects: After matching, the Average Treatment Effect on the Treated (ATT) is calculated using the matched samples, which reflects the impact of pet ownership on attitudes toward animal welfare and consumption intentions. The estimation formula for ATT is:

ATT = E{Y_1i_ − Y_0i_|D_i_ = 1} = E[E{Y_1i_ − Y_0i_|D_i_ = 1, p(X)_i_}] = E[E{Y_1i_|D_i_ = 1, p(X)_i_} − E{Y_0i_|D_i_ = 0, p(X)_i_}|D_i_ = 1]

where Y_1_ and Y_0_ represent the outcome differences on the dependent variables (attitudes toward animal welfare and behavioral intentions) for individuals from households with and without pets, respectively.

All statistical analyses were conducted using Stata 17.0 software. A significance level of 0.1 was used, which allows for detecting marginally significant effects, common in social science research where subtle effects may be important. After matching the samples using PSM, paired sample *t*-tests were employed to compare the mean differences between the treatment and control groups on the dependent variables (attitudes toward animal welfare and consumption intentions). The *t*-tests assess whether significant differences exist between pet-owning and non-pet-owning students while accounting for covariates. Robust standard errors were applied to account for potential heteroscedasticity, ensuring the reliability of the results. The statistical tests and significance levels are explicitly reported in the results section and referenced in Table 2.

## 4. Empirical Results and Econometric Analysis

### 4.1. Descriptive Findings

Table 2 presents a descriptive analysis comparing differences in animal welfare attitudes and consumption intentions between university students from households with pets and those from households without pets. The results indicate that students from households with pets exhibit significantly higher levels of animal empathy, perceptions of animal welfare, willingness to purchase animal welfare certified products, and willingness to pay a premium for animal welfare labels. Specifically, the mean animal empathy scores for students from pet-owning households were 0.186 points higher than those from non-pet-owning households, indicating a greater likelihood of moderate to high empathy levels among pet owners (*p* < 0.01). Regarding perceptions of animal welfare, the proportion of students with high perceptions is 10.1% higher for those from pet-owning households (*p* < 0.01), suggesting that pet ownership may enhance students’ sensitivity to and understanding of animal welfare issues.

In terms of consumption intentions, pet ownership also plays a significant role. The proportion of students with a high willingness to purchase animal welfare certified products is 8.2% higher among those from households with pets (*p* < 0.05), indicating that pet ownership not only influences attitudinal factors but also has a tangible impact on consumption behaviors. Additionally, students from pet-owning households are willing to pay an average of CNY 0.137 more per bottle (*p* < 0.1) for animal welfare certified products compared to those from households without pets, further demonstrating the influence of pet ownership on consumption choices. Overall, these findings suggest that pet ownership significantly affects university students’ attitudes toward animal welfare and their consumption intentions, with all differences being statistically significant.

### 4.2. Results of Average Treatment Effect on the Treated (ATT)

Table 3 presents the Average Treatment Effect on the Treated (ATT) of pet ownership on Chinese university students’ attitudes toward animal welfare and their consumption intentions. The results from kernel matching show that students from households with pets exhibit significantly more positive attitudes and behavioral intentions related to animal welfare across all variables. Specifically, the animal empathy scores for students from pet-owning households were 0.178 points higher than those from non-pet-owning households (*p* < 0.01). Additionally, the proportion of students with high perceptions of animal welfare is 9.5% higher (*p* < 0.01), while the proportion of those with a strong willingness to purchase animal welfare certified products is 8.2% higher (*p* < 0.05). Regarding the willingness to pay a premium for animal welfare labels, students from pet-owning households are willing to pay an additional CNY 0.139 per bottle of milk with an animal welfare label (*p* < 0.1).

To ensure the robustness of the results, we also conducted analyses using radius matching and nearest neighbor matching. While there are slight variations in the absolute values of the average treatment effects, the direction, magnitude, and significance levels of the coefficients remain consistent across all methods. This consistency further supports the positive impact of pet ownership on students’ attitudes and consumption intentions regarding animal welfare. These findings demonstrate that the positive effect of pet ownership is robust, regardless of the matching method employed.

### 4.3. Matching Effectiveness Test

**Figure A1:**
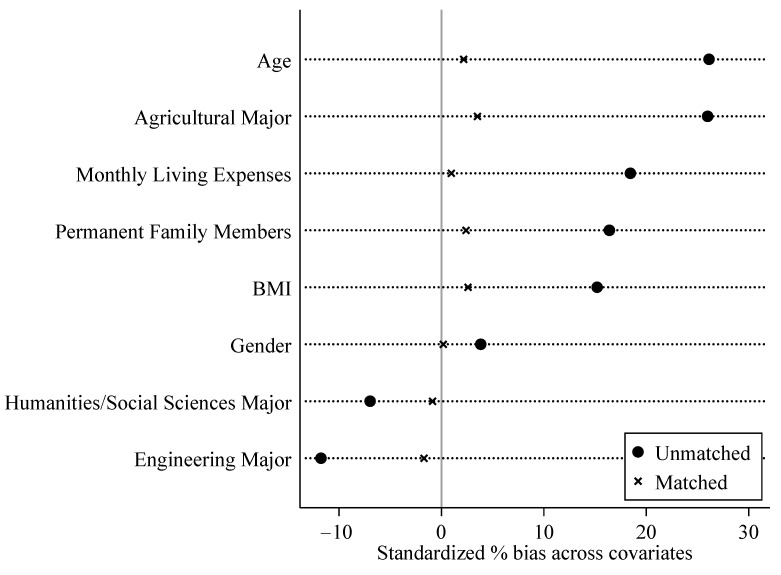
Common range of propensity scores.

**Figure A2:**
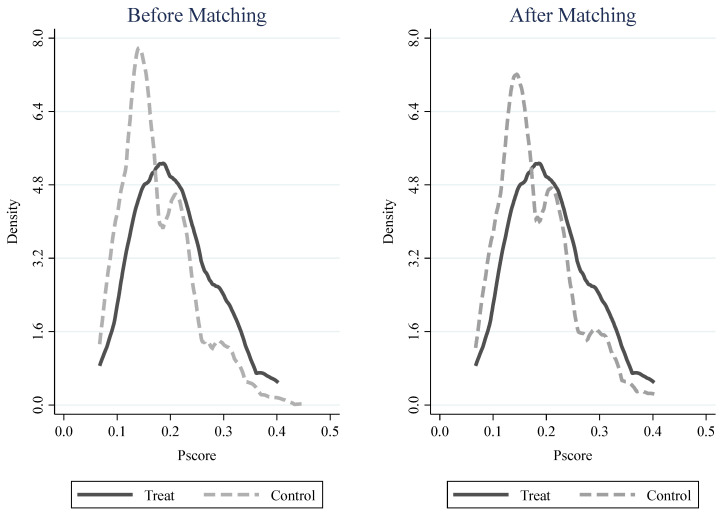
Kernel density comparison before and after matching.

A key assumption of the PSM method is that there should be significant differences in propensity scores between the treatment and control groups before matching, and these differences should be substantially reduced or eliminated after matching. To assess the effectiveness of the matching process, kernel matching is used as an illustrative example. As shown in Figure A1, most observations fall within the common range of propensity scores, indicating minimal sample loss during the matching process. Figure A2 presents the kernel density comparison before and after matching, where the two curves are notably more aligned post-matching. Specifically, there is a visible reduction in the peak and a shrinking tail in the control group, demonstrating that the differences in propensity scores between the treatment group (students with pet ownership) and the control group (students without pet ownership) have significantly decreased.

**Figure A3:**
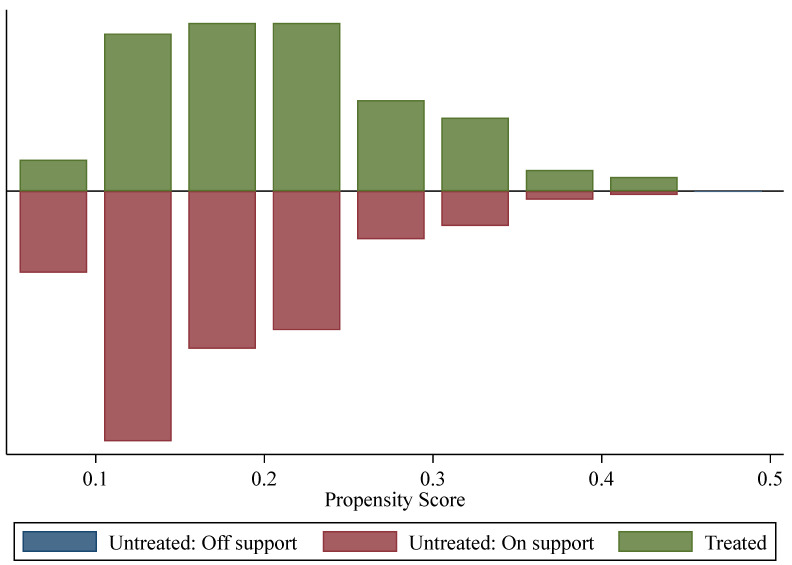
Common range of values for the propensity score. Group assignment after matching: Untreated: Off support (blue), N = 1; Untreated: On support (red), N = 932; Treated (all on support, green), N = 207. The “Untreated: Off support” group is not visible due to its minimal size. Excluding such small groups in PSM is standard practice to ensure representativeness and comparability, minimizing potential bias from sample imbalance or matching issues.

Table A1 provides a detailed comparison of the differences in covariates between the treatment and control groups before and after matching. Prior to matching, significant differences were observed between the two groups in variables such as age, BMI, agricultural major, living expenses, and the number of permanent family members. However, after matching, these significant differences were eliminated, and variables that originally showed no significant differences became even less significant post-matching. Figure A3 visually illustrates the changes in standardized biases of the control variables before and after matching, demonstrating a substantial reduction in bias following the matching process. Overall, the matching process was effective, enhancing the credibility and robustness of the study’s findings.

Table A2 further tests the reliability of the PSM results. The average standardized bias of the control variables before matching was 46.4%, while after matching, this value dropped to between 5.4% and 8.6%, well below the 20% threshold typically used for balance testing. Additionally, the pseudo R² decreased from 0.033 before matching to 0.001 after matching, and the LR statistic dropped significantly from 35.98 before matching to a range of 0.30 to 0.76 after matching. These results demonstrate that the application of PSM effectively reduced the differences in control variables between the treatment and control groups, thereby mitigating estimation bias caused by sample self-selection.

## 5. Discussion

As global attention to animal welfare issues intensifies, consumer attitudes and behavioral intentions have emerged as key drivers in the development of the animal welfare certified product market [42]. Within this context, the present study investigates how pet ownership influences Chinese university students’ attitudes toward animal welfare and their consumption intentions. Using the PSM method, the empirical results reveal that living in a household with pets significantly enhances students’ animal empathy, perceptions of animal welfare, and their willingness to purchase animal welfare certified products and pay a premium for them. These findings offer empirical support for understanding animal welfare awareness in the Chinese context and highlight the role of pet ownership in promoting ethically driven consumer behavior.

Regarding the impact of pet ownership on attitudes toward animal welfare, our findings align with the conclusions of Auger and Amiot [23], who suggested that living in a household with pets can enhance individuals’ emotional resonance and increase their attention to animal welfare. Among the specific group of Chinese university students, we found that those from pet-owning households are more likely to exhibit higher levels of animal empathy. This suggests that the emotional bond between pets and their owners is effective not only in Western cultural contexts but also in a cultural context like China. This finding further extends Serpell and Paul’s [22] “pets as ambassadors” hypothesis, indicating that pet ownership can influence individuals’ perceptions and attitudes toward other animals. Additionally, the connection between pet ownership and animal welfare perceptions proposed by McKendree et al. [34] was confirmed in our study. University students from pet-owning households demonstrate not only heightened emotional resonance but also a deeper perceptual understanding of animal welfare issues, reflecting a more comprehensive awareness of animal rights and welfare.

The impact of pet ownership on consumption intentions related to animal welfare is another key finding of this study. To assess willingness to pay a premium for animal welfare certified products, we used milk as an example due to its familiarity and accessibility. Students from pet-owning households exhibit significantly higher willingness to purchase animal welfare certified products than those without pets. This aligns with the findings of Pirsich et al. [25] and Pearce et al. [26], which suggest that pet ownership can shape ethical consumption behavior. Additionally, our results further support previous research [1], showing pet ownership positively influences both purchase intentions and willingness to recommend animal welfare products. In our study, the increased willingness to pay a premium for products like animal welfare labeled milk highlights the role of pet ownership in enhancing consumer behavior. This aligns with Pettersson et al. [47], who demonstrated that animal welfare labels can significantly influence consumer decisions. Our findings extend this by showing that pet ownership amplifies the effect, suggesting that it motivates consumers to support animal welfare certified products.

Building on the existing literature on animal welfare, this study is the first to empirically examine the impact of pet ownership on university students’ attitudes and consumption intentions toward animal welfare within the Chinese cultural context. Platto et al. [28] previously highlighted that the influence of pet ownership on attitudes toward animal welfare may vary across different social and cultural settings. While awareness of animal welfare is more established in Western societies, it is still emerging in China. In Guangdong Province—a region known for its economic development and openness to global ideas—university students exhibit attitudes and behaviors that align with international trends. Our empirical data demonstrate that pet ownership significantly enhances students’ emotional engagement and shapes their consumption behavior. Guangdong’s socio-economic characteristics, including its exposure to global trends, may partly explain the strong openness toward animal welfare observed among students. This finding suggests that as animal welfare continues to gain prominence in China, pet ownership could play a crucial role in shifting consumer behavior and fostering ethical consumption.

While this study focuses on the relationship between pet ownership and individual attitudes and consumption behavior, the implications extend beyond pet-owning households. The connection between pet ownership and ethical values highlights the need for coordinated efforts across education, business, and government sectors to promote animal welfare and sustainable consumption. Achieving widespread animal welfare awareness requires a multi-sectoral approach. Based on these insights, we propose the following recommendations to enhance the impact of animal welfare awareness and foster a culture of ethical consumption and responsibility. First, educational institutions should integrate animal ethics and social responsibility into their curricula, helping students build a comprehensive set of moral values. Second, businesses should leverage the growing trend of ethical consumption, particularly among younger consumers, by promoting products that meet animal welfare standards and increasing the visibility of animal welfare certifications. Lastly, policymakers should institutionalize animal welfare by implementing certification systems and offering incentives for companies that adopt higher welfare standards. These combined efforts will elevate societal awareness of animal welfare, promote responsible consumption, and support sustainable development.

Despite the valuable insights offered, this study has certain limitations. First, the data for this study are drawn exclusively from university students in Guangdong Province. While Guangdong reflects a degree of economic development and openness to ideas, the external validity of the results may be limited when applied to other regions or demographic groups. Future research could expand to additional regions to assess whether these findings have broader applicability. Second, while university students play a significant role in social change, their behaviors and attitudes may not fully capture the perspectives of other social groups regarding animal welfare issues. Thus, future studies should consider incorporating a more diverse population to enhance the generalizability of the research. Third, this study utilizes cross-sectional data. Although the PSM method helps address endogeneity concerns, it does not account for the long-term effects of pet ownership on individual attitudes and behavioral intentions from a dynamic perspective. Future research could employ longitudinal data to further improve the accuracy of causal inferences. Lastly, this study did not collect detailed information about the nature of the relationship between respondents and their household pets, such as the extent of their involvement in pet care, the type of pet, or the emotional bond between the respondents and the pets. Future studies could investigate the quality of pet ownership, including emotional commitment, attachment, and the depth of interaction with pets, to gain deeper insights into how these factors influence attitudes toward animal welfare and consumption behaviors.

## 6. Conclusions

As animal welfare gains increasing global importance, understanding the factors that influence individual attitudes and consumption intentions in this area is crucial for promoting greater awareness and practice. Using the PSM method, this study empirically examined the impact of pet ownership on the animal welfare attitudes and consumption intentions of Chinese university students. The findings demonstrate that pet ownership significantly enhances students’ animal empathy, perceptions of animal welfare, willingness to purchase animal welfare certified products, and willingness to pay a premium for such products. Specifically, university students from households with pets exhibit more positive attitudes and behaviors, including greater emotional resonance with animals, a deeper understanding of animal welfare, and more favorable consumption behaviors. Compared to students from households without pets, they are more likely to endorse the concept of animal welfare and are willing to pay a higher premium for animal welfare certified products. It is evident that pet ownership is not simply a routine activity, but one that has a meaningful impact on individuals’ views of animal welfare across emotional, perceptual, and consumption-related dimensions.

This finding provides new empirical evidence for related theoretical research and offers valuable insights for policymakers and businesses. Based on these results, future education and advocacy efforts should focus on promoting concepts such as animal ethics, social responsibility, and sustainable development to achieve broader societal recognition and practice of animal welfare. This study not only highlights the significant impact of pet ownership on various dimensions of animal welfare views through empirical analysis, but also addresses endogeneity issues, to some extent, by using the PSM method, thereby strengthening the study’s causal inference capabilities. This approach improves upon the limitations of previous studies, which often explored only simple correlations, and provides more robust empirical support for theoretical research. However, this study is limited by the lack of detailed data on the depth of interaction between respondents and their household pets, as well as the regional and demographic scope of the sample. Future research could expand to include a broader range of social groups and regions, incorporate longitudinal data, and conduct mechanism analyses to further investigate the long-term impact of pet ownership and the quality of interaction on animal welfare attitudes and consumption behavior. By broadening the scope and deepening the analysis, future research can offer richer theoretical and practical guidance for advancing animal welfare in China and beyond.

## Figures and Tables

**Table 1:** Definitions and measurement of control variables.

Variable Name	Definition and Measurement
Gender	Female = 1, Male = 0
Age	Above average age = 1, Below average age = 0
BMI	≥24.00 = 2, 18.50–23.99 = 1, ≤18.50 = 0
Agricultural Major	Yes = 1, No = 0
Engineering Major	Yes = 1, No = 0
Humanities/Social Sciences Major	Yes = 1, No = 0
Monthly Living Expenses	Above average living expenses = 1, Below average = 0
Permanent Family Members	Above average number of family members = 1, Below average = 0

**Table 2:** Descriptive comparison of pet ownership on university students’ animal welfare attitudes and consumption intentions.

Variable	Full Sample	Households With Pets	Households Without Pets	Difference
Animal Empathy	1.152 (0.747)	1.304 (0.737)	1.118 (0.745)	0.186 ***
Perceptions of Animal Welfare	0.444 (0.497)	0.527 (0.501)	0.426 (0.495)	0.101 ***
Willingness to Purchase Animal Welfare Certified Products	0.290 (0.454)	0.357 (0.480)	0.275 (0.447)	0.082 **
Willingness to Pay a Premium for Animal Welfare Labels	3.617 (0.960)	3.730 (0.963)	3.592 (0.959)	0.137 *

Note. The values represent the means for each group, with standard deviations shown in parentheses. The “Difference” column reports the mean difference between students from households with pets and those from households without pets. Positive values indicate higher mean scores for students from households with pets. Statistical significance of the differences was assessed using t-tests: *** *p* < 0.01, ** *p* < 0.05, * *p* < 0.1.

**Table 3:** Average treatment effect of pet ownership on university students.

Variable	Kernel Matching		Radius Matching		Nearest Neighbor Matching	
	ATT	Std. Error	ATT	Std. Error	ATT	Std. Error
Animal Empathy	0.178 ***	0.050	0.169 ***	0.061	0.178 ***	0.062
Perceptions of Animal Welfare	0.095 ***	0.037	0.085 **	0.040	0.112 **	0.047
Willingness to Purchase Animal Welfare Certified Products	0.082 **	0.037	0.083 **	0.040	0.106 **	0.050
Willingness to Pay a Premium for Animal Welfare Labels	0.139 *	0.075	0.148 **	0.075	0.152 *	0.088

Note. Standard errors are shown in parentheses. *** *p* < 0.01, ** *p* < 0.05, * *p* < 0.1.

## Data Availability

The datasets generated during and/or analyzed during the current study are available from the corresponding author on reasonable request.

## Appendix A

**Table A1:** Balance test results for covariates before and after matching.

Variable	Umatched	Mean		%Bias	%Reduct |Bias|	*t*-Test	
	Matched	Treated	Control			*t*	*p* > |*t*|
Gender	U	0.623	0.605	3.8	95.6	0.50	0.619
	M	0.623	0.622	0.2		0.02	0.986
Age	U	0.589	0.460	26.1	91.7	3.39	0.001
	M	0.589	0.576	2.2		0.22	0.825
BMI	U	1.053	0.964	15.2	82.9	1.97	0.049
	M	1.053	1.042	2.6		0.26	0.792
Agricultural Major	U	0.300	0.189	26.0	86.5	3.57	0.000
	M	0.300	0.285	3.5		0.33	0.739
Engineering Major	U	0.213	0.263	−11.8	85.5	−1.50	0.135
	M	0.213	0.221	−1.7		−0.18	0.858
Humanities/Social Sciences Major	U	0.222	0.252	−7.0	87.5	−0.90	0.371
	M	0.222	0.227	-0.9		−0.09	0.928
Living Expenses	U	0.488	0.397	18.4	94.9	2.42	0.016
	M	0.488	0.485	0.9		0.10	0.924
Permanent Family Members	U	0.420	0.341	16.4	85.4	2.16	0.031
	M	0.420	0.407	2.4		0.24	0.811

**Table A2:** Balance test results for control variables before and after matching using different matching methods.

Matching Method	Pseudo R²	LR Statistic	Standardized Bias (%)
Before Matching	0.033	35.98	46.4
Kernel Matching	0.001	0.30	5.4
Radius Matching	0.001	0.68	8.1
Nearest Neighbor Matching	0.001	0.76	8.6

## Appendix B

Preliminary Question

Do you regularly purchase milk?

○ Yes
○ No (End of the survey)

I. Basic Information

What is your gender?

○ Male
○ Female

How old are you? _____ years old

Where do you live?

○ Urban
○ Rural

What is your educational background?

○ Primary school or below
○ Junior high school
○ Senior high school/Vocational school
○ College
○ Bachelor’s degree
○ Master’s degree or higher

What is your occupation?

○ Government/Institution/Public Servant
○ Company/Corporate Employee
○ Professional (e.g., Education, Healthcare)
○ Self-employed/Freelancer
○ Farmer/Rural laborer
○ Student
○ Unemployed or Retired
○ Other (please specify)

What is your major?

○ Agricultural
○ Engineering
○ Humanities/Social Sciences
○ Other

What is your monthly living expense? _____ CNY

What is your height? _____ cm

What is your weight? _____ kg

How many people are there in your household? _____ persons

Does your household keep pets?

○ Yes
○ No

II. Attitudes Toward Animal Welfare and Consumption Intentions

How do you evaluate the following statement: “I feel upset every time I see animals being abused or in pain”?

○ Strongly disagree
○ Disagree
○ Neutral
○ Agree
○ Strongly agree

Please evaluate the following statements based on your personal experience.

**Item (Statement)**	**Strongly Disagree**	**Disagree**	**Neutral**	**Agree**	**Strongly Agree**
Since farm animals will ultimately be slaughtered for consumption, their welfare doesn’t matter.					
Human welfare has yet to be met, and it’s not time to consider animal welfare.					
The cost of welfare-oriented farming is too high and not suitable for our country’s reality.					
Some welfare farming practices are merely due to farmers’ curiosity and novelty, or for commercial selling points.					

If there is animal welfare-certified milk available on the market, would you be willing to purchase it?

○ Definitely not
○ Probably not
○ Might consider
○ Probably will
○ Definitely will

III. Choice Experiment

People’s purchasing decisions in hypothetical scenarios may differ from their real-life behavior. This difference is called hypothetical bias. In hypothetical scenarios, people often overestimate their willingness to pay for products, whereas in real-life situations, they may reconsider based on affordability.

In this section, you will be presented with six sets of milk with different qualities. These types of milk are available in supermarkets where you usually shop, with prices ranging from 2.8 to 8.8 CNY per bottle. For each set, you can choose one of the two options or neither. It is important that you make your choices as if you were selecting milk in your usual shopping scenario.

Below is some additional information about the milk:

- Brand: Refers to the domestic or international brand.
- Shelf Life: The period during which the pre-packaged milk maintains its quality under the specified storage conditions.
- Protein Content: The amount of protein per 100 mL of milk.
- Label: Indicates whether the milk is produced following animal welfare or organic certification standards.
- Price: The price per 250 mL bottle.

Before starting, please provide your birth month:

○ January or July
○ February or August
○ March or September
○ April or October
○ May or November
○ June or December

Due to space constraints, only the first block of the experiment (for respondents born in January and July) is presented below:

1/10/36

1/12/36

1/16/36

1/17/36

1/23/36

1/25/36
